# *Notes from the Field:* Unintentional Fentanyl Overdoses Among Persons Who Thought They Were Snorting Cocaine — Fresno, California, January 7, 2019

**DOI:** 10.15585/mmwr.mm6831a2

**Published:** 2019-08-09

**Authors:** Patil Armenian, Jeffrey D. Whitman, Adina Badea, Whitney Johnson, Chelsea Drake, Simranjit Singh Dhillon, Michelle Rivera, Nicklaus Brandehoff, Kara L. Lynch

**Affiliations:** ^1^Department of Emergency Medicine, University of California San Francisco, Fresno, California; ^2^Department of Laboratory Medicine, University of California San Francisco, San Francisco, California; ^3^Fresno County Department of Public Health, Fresno, California.

On January 7, 2019, three patients arrived at the Community Regional Medical Center emergency department in Fresno, California, after snorting (i.e., nasally insufflating) white powder they thought was cocaine. One (patient A) was in cardiac arrest, and two (patients B and C) had opioid toxidrome (miosis, respiratory depression, and depressed mental status) (Table). After spontaneous circulation was reestablished in patient A, he was admitted to the intensive care unit, where he was pronounced brain-dead 3 days later. Patients B and C responded to naloxone, but repeated dosing was required to maintain respiratory status. Routine urine drug screens, which do not include testing for synthetic opioids such as fentanyl, were negative for opioids for all three patients. This finding, in combination with opioid toxidrome requiring repeated doses of naloxone, caused the medical toxicology team to be suspicious of an unintentional synthetic opioid exposure, and they notified the Fresno County Department of Public Health (FCDPH). After discussion with law enforcement the following day, a fourth patient (patient D) was identified in neighboring Madera County. Patient D was in cardiac arrest when emergency medical services arrived, and she was pronounced dead at the scene. Blood and urine specimens for patients A, B, and C were analyzed using liquid chromatography quadrupole time-of-flight mass spectrometry* for 13 fentanyl analogs and metabolites,^†^ one novel synthetic opioid (U-47700), and 157 other drugs and metabolites. Results confirmed fentanyl without fentanyl analogs or other novel synthetic opioids.

**TABLE:** Demographic characteristics, naloxone administration characteristics, toxicology results,* and outcomes of four patients with fentanyl overdoses — Fresno and Madera Counties, California, January 7, 2019

Characteristic	Patient			
	A	B	C	D
**Age group (yrs)**	30–39	20–29	20–29	30–39
**Sex**	Male	Male	Male	Female
**Provider/Route and naloxone dose (mg)**				
EMS/Intranasal	N/A	3.0	2.0	N/A
ED/Intravenous	N/A	1.0	0.4, 1.0	N/A
**Outcome**	ICU, brain death 3 days later	TU, discharge on day 2	TU, discharge on day 2	Pronounced dead at scene
**Serum drug levels (ng/mL)**	fentanyl 2.5; norfentanyl 0.4	fentanyl 5.3; norfentanyl 0.6	fentanyl 4.3; norfentanyl <0	N/A
**Other substances detected in serum**	Cotinine	Methamphetamine, amphetamine, cotinine	none	Fentanyl, norfentanyl^†^

**Abbreviations**: ED = emergency department; EMS = emergency medical services; ICU = intensive care unit; N/A = not applicable; TU = telemetry unit.

* Except for patient D, testing was performed on blood specimens obtained upon initial hospital evaluation; for patient D, testing was performed on postmortem blood specimen. Testing for patients A, B, and C was done at the University of California San Francisco Clinical Laboratory, Zuckerberg San Francisco General Hospital using liquid chromatography quadrupole time-of-flight mass spectrometry.

^†^ Included as other because quantitative levels could not be determined.

After notification of the initial three cases, a multiagency response was implemented by FCDPH; Fresno County Sheriff-Coroner’s Office; Fresno Police Department; Fresno County Department of Behavioral Health; Community Regional Medical Center; University of San Francisco-Fresno; and the Drug Enforcement Administration. Initial actions included 1) disseminating a news release targeting the media and other emergency departments; 2) holding a multiagency press conference; 3) conducting media interviews; 4) informing law enforcement, prehospital providers, and the public about naloxone distribution and use; 5) educating persons on the proper disposal of old or new but unused medications through the Fresno County Department of Behavioral Health/California Health Collaborative drop-off containers^§^; and 6) publicizing the California Central Valley Opioid Safety Coalition webpage,^¶^ which provides information about naloxone and substance use disorders.

On January 12, 2019, a similar drug overdose incident was reported in Chico, California, in which postmortem toxicology testing for one person confirmed fentanyl (*1*). Fourteen other persons at the same event were hospitalized with opioid toxidrome and later released. They reported thinking they were snorting cocaine,** but confirmatory toxicology results are unavailable. Fresno, Madera, and Chico are located along the same state highway (CA-99) corridor.

Death rates involving cocaine increased by approximately one third during 2016–2017. In 2017, nearly three fourths of cocaine deaths also involved opioids, with the arrival of synthetic opioids driving much of this increase (*2*). Mixing of drugs is a phenomenon being detected at a national level, with some variation across regions (*2*). There have been other reports of outbreaks caused by fentanyl disguised as cocaine among opioid-naïve populations in New Haven, Connecticut, and Philadelphia, Pennsylvania (*3*,*4*). These reports indicate similar exposures to low serum fentanyl concentrations (*3*,*4*) and also describe the need for multiple naloxone doses for effective reversal. In British Columbia, Canada, furanyl fentanyl caused an outbreak in patients who thought they were smoking crack cocaine (*5*). Fentanyl is likely underdetected because it is not routinely included on hospital urine immunoassays and it is useful to know the limitations of an institution’s screening techniques. Targeted and untargeted analyses are necessary to detect fentanyl, fentanyl analogs, and other novel synthetic opioids (*6*). Traditional toxicology testing is targeted at specific known drugs, whereas liquid chromatography quadrupole time-of-flight mass spectrometry analysis can either detect an unexpected drug from patient or drug product specimens or match an unknown molecular weight on the spectra with a specific chemical formula to identify a novel drug.

In the days following the multiagency press conference, FCDPH disseminated a California Health Alert Network message to approximately 700 Fresno County providers about free online medication-assisted treatment waiver training and encouraged use of the Controlled Substance Utilization Review and Evaluation System prescription drug–monitoring program for opioid users.^††^ Local emergency departments continued to focus on referring persons using drugs other than opioids (e.g., cocaine) to substance use disorder treatment as indicated. FCDPH continues to monitor potential fentanyl overdose cases and work with the Fresno County Department of Behavioral Health, medical providers, and the Fresno County Sheriff-Coroner’s Office to educate and warn the public about the risks of street drugs. On January 24, a suspect was charged with two counts of distributing fentanyl resulting in death, related to the overdoses in Fresno and Madera counties described in this report.^§§^ This multiagency response was key to disseminating information to the public and other health providers about the outbreak and about naloxone distribution and use. Efforts to better understand the nature of substance use and co-involvement of different drug classes is needed for tailored prevention and response strategies.

## Footnotes

* Analysis conducted at the University of California San Francisco Clinical Laboratory, Zuckerberg San Francisco General Hospital.

† Fentanyl, norfentanyl (metabolite), butyryl fentanyl, acetylfentanyl, 3-methylfentanyl, beta-hydroxythiofentanyl, furanyl fentanyl, para-fluorofentanyl, fluorobutyryl fentanyl, carfentanil, acrylfentanyl, tetrahydrofuranfentanyl, cyclopropyl fentanyl.

§ https://healthcollaborative.org/lock-it-up-project/.

¶ http://centralvalleyopioidsafety.org/.

** http://actionnewsnow.com/content/news/Overdose-Victim-Family-Members-Speak-Out-About-Incident--504403741.html.

††
https://oag.ca.gov/cures .

§§ https://www.dea.gov/press-releases/2019/01/25/fresno-man-charged-two-counts-distributing-fentanyl-resulting-death.
